# Vitamin D Deficiency and Vasovagal Syncope in Children and Adolescents

**DOI:** 10.3389/fped.2021.575923

**Published:** 2021-02-25

**Authors:** Qingyou Zhang, Yan Sun, Chunyu Zhang, Jianguang Qi, Junbao Du

**Affiliations:** Department of Pediatrics, Peking University First Hospital, Beijing, China

**Keywords:** children and adolescents, vasovagal syncope, vitamin D, heart rate variability, autonomic nervous function

## Abstract

**Aims:** To investigate the association of vitamin D deficiency with cardiovascular autonomic nervous system function in children and adolescents with vasovagal syncope (VVS).

**Methods:** This study recruited 76 pediatric patients with VVS and 15 healthy children. The 25-hydroxyvitamin D levels in serum among the participants were evaluated. Heart rate variability analysis including SDNN, rMSSD, and SDANN was tested in patients with VVS. The correlation between indices of time-domain analysis and serum vitamin D status of the children with VVS was investigated.

**Results:** In this work, 25-hydroxyvitamin D levels in serum among VVS cases remarkably decreased compared with those among healthy controls (48.76 ± 19.25 vs. 67.62 ± 15.46 nmol/L, *p* < 0.01). The vitamin D deficient patients with VVS exhibited a lower rMSDD value compared to the non-deficient group with VVS (45.56 ± 16.87 vs. 61.90 ± 20.38 ms, *p* < 0.001, respectively). Pearson correlation analysis indicated that serum 25-hydroxyvitamin D levels had positive correlation with rMSDD values (*r* = 0.466, *p* < 0.001).

**Conclusions:** As suggested by our data, VVS children and adolescents with vitamin D deficiency may have cardiac autonomic dysfunction and cardiac vagal tone decreases with the reduction in vitamin D level.

## Introduction

Syncope is a common occurrence in the pediatric population. Before the end of adolescence, about 15% of children and adolescents experience at least one episode of syncope (1, 2). The most common etiology of syncope in pediatric patients is vasovagal syncope (VVS) (3). Recurrence of VVS affects physical, psychological, and psychosocial activities of life, leading to impaired quality of life. However, the exact pathogenesis of VVS is currently unknown (4).

It has been reported that orthostatic intolerance including VVS in children is associated with many malnutrition diseases, including low iron storage (5), vitamin B12 deficiency (6), vitamin B1 deficiency (7), and others. Vitamin D is one of fat-soluble nutritive molecules that is crucial for calcium and phosphorus homeostasis. However, recent research has identified vitamin D as a prohormone with a wide range of actions in human diseases, particularly in the cardiovascular system (8, 9). It is related to the modulation of blood pressure, heart functions, coronary atherosclerosis, and calcification (8, 9). Some studies show that vitamin D can also regulate the cardiovascular autonomic tone (10, 11). Hypovitaminosis D has been associated with the disturbance of the cardiovascular autonomic system contributing to the development of an array of diseases including hypertension, orthostatic hypotension, and postural orthostatic tachycardia syndrome (10–13).

VVS is one of the most common diseases in the cardiovascular autonomic system in youngsters. In adult VVS patients, Usalp et al. (14) recently reported that serum vitamin D levels were low in patients with syncope, especially in patients diagnosed with VVS by HUTT test. However, the relationship between hypovitaminosis D and the cardiac autonomic nervous function state in children with VVS have not been studied. This research was undertaken for investigating correlation of vitamin D contents in serum with cardiac autonomic function in the pathogenesis and development of VVS in youngsters.

## Methods

This study included 76 patients (7–18 years old) with VVS. These patients were referred to Pediatric Cardiology, Peking University First Hospital (China) for an evaluation of unexplained syncope between May 2018 and November 2019. Routine evaluation in all of the patients was performed including a thorough investigation of their medical history, complete physical examination, 12-lead standard electrocardiogram (ECG), head-up tilt test and basic laboratory examinations. Fifteen healthy control subjects were included in this study and they were referred to our department for a cardiovascular assessment for an innocent murmur in the same months of the year as the VVS-group. The criteria for VVS diagnosis were determined based on a previously described protocol (3). Patients with neurologic, psychiatric, cardiovascular disorders including hypertension, and obesity were excluded from the study. All enrolled patients (with VVS), and their parents signed informed consent for the tilt test and the blood tests; the control subjects and their parents signed for the blood tests. The Ethics Committee of Peking University First Hospital approved this study.

### Study Design

For investigating the association of vitamin D levels in serum with VVS, we compared VVS cases and control subjects for their serum contents of vitamin D. According to their serum levels, patients with VVS were then classified in deficient vitamin D or non-deficient group. To investigate how vitamin D deficiency affected the cardiac autonomic function between these two groups, we further compared time-domain parameters of heart rate variability (HRV) and examined the association of serum vitamin D contents with the indices of time-domain analysis in VVS patients.

### Serum Vitamin D Assessment

Vitamin D from diet and skin exposure to sunlight can be assessed based on serum 25-hydroxyvitamin D (25(OH)D) level. So, vitamin D contents in serum (chemiluminescence immunoassay) were determined by detecting the 25(OH)D contents in serum. Each specimen was collected following 12 h of overnight fasting. According to recent clinical guidelines (15), vitamin D deficiency is defined as the 25(OH)D content in serum of <50 nmol/L.

### Heart Rate Variability

Twenty-four-hour electrocardiogram (Holter) was examined for all enrolled patients. In order to reduce the influence of various tests, there was no overlap between 24-h Holter ECG and other cardiovascular tests. Holter ECG recordings of each patient were of good quality, as none of the patients experienced frequent premature contractions (less or equal to one contraction per hour). HRV was analyzed with an automatic Holter analysis system (DMS version 12.5, USA). Premature beats and artifacts of ECG were adjusted by interpolation with the previous and next successive heartbeat. HRV time-domain analysis was performed following calibration. The evaluation standards, physiological explanation, and biological signal processing algorithms were done following the guidelines from the North American Society of Pacing and Electrophysiology, as well as the Task Force of the European Society of Cardiology (16). Besides, HRV indices, including standard deviations (SDs) for all normal to normal heart rate intervals over 24 h (SDNN), percentage of differences between adjacent RR intervals that are >50 ms (pNN50), root-mean-square difference in the interval between two normal heart rates (rMSSD) and SDs for 5 min average interval between two normal heart rates (SDANN), were calculated. SDNN depends on a change of the overall autonomic nervous system activity of the heart. rMSSD is an HRV parameter that shows the parasympathetic activity of the heart while SDANN shows the sympathetic activity of the heart (16).

### Statistical Methods

SPSS18.0 (SPSS Inc., Chicago, IL, USA) was adopted for all statistical analyses. All measurement variables were presented in the manner of mean ± SD. Continuous variables with normal distribution were analyzed by independent *t*-test, whereas chi-squared test was adopted for categorical variables, and Pearson correlation coefficient was determined to examine the association of 25(OH)D content in serum with the rMSDD value. A difference of *p* < 0.05 indicated statistical significance.

## Results

### Demographic Characteristics and Serum Vitamin D Status of Participants With VVS

A total of 76 youngsters aged 7–18 years participated in this study. Among those patients, 21 (27.6%) were male and 55 (72.4%) were female. The mean BMI in this cohort was 18.5 ± 2.3 kg/m^2^. Compared with healthy control group, age, sex, BMI, baseline BP, and baseline heart rate did not differ in children and adolescents with VVS. The average 25(OH)D content in serum among the test patients was 48.76 ± 19.25 nmol/L. Compared with healthy control group, VVS children and adolescents had significantly low 25(OH)D content in serum. Using serum level of 25(OH)D <50 nmol/L as standard for hypovitaminosis D, there were 60.5% of hypovitaminosis D in test patients, significantly higher than the healthy control group (Table 1).

**Table 1:** Demographic and clinical characteristics of patients and control subjects.

**Characteristics**	**VVS group**	**Control group**	***P*-value**
Cases, n	76	15	
Age, years	12 ± 2	11 ± 2	0.296
BMI, kg/m^2^	18.5 ± 2.3	17.4 ± 1.4	0.07
Females/males, n	55/21	9/6	0.364
Baseline systolic BP, mmHg	106.5 ± 8.0	107.3 ± 8.4	0.747
Baseline diastolic BP, mmHg	63.8 ± 6.3	65.6 ± 10.4	0.386
Baseline hear rate, beats/min	78.3 ± 10.0	77.9 ± 8.6	0.884
25(OH)D level, nmol/L	48.76 ± 19.25	67.62 ± 15.46	0.001
Children with vitamin D deficiency, n (%)	46 (60.5)	2 (13.3)	0.001

*BMI, body mass index; BP, blood pressure*.

### Associations of Vitamin D Value in Serum With HRV Indices in Youngsters With VVS

Table 2 illustrates patient demographic features according to the 25(OH)D status. Differences in 25(OH)D level between vitamin D deficient and non-deficient groups showed no significance with regard to sex, age, BMI, baseline blood pressure, or baseline heart rate. However, in the serum vitamin D deficient test patients exhibited a lower rMSDD value than the non-deficient test group (45.56 ± 16.87 vs. 61.90 ± 20.38 ms, *p* < 0.001, respectively). The other time-domain parameters, including SDNN, pNN50, and SDANN, did not differ between the two VVS groups.

**Table 2:** Characteristics and HRV indices of time-domain analysis in children and adolescents with VVS based on 25-hydroxyvitamin D status.

**Characteristics**	**Deficient group**	**Non-deficient group**	***P*-value**
Cases, n	46	30	
Age, years	12 ± 2	12 ± 2	0.237
BMI, kg/m^2^	18.9 ± 2.4	18.0 ± 2.3	0.084
Females/males, n	33/13	22/8	0.879
Baseline systolic BP, mmHg	107.6 ± 8.2	104.9 ± 7.7	0.156
Baseline diastolic BP, mmHg	64.4 ± 6.2	62.9 ± 6.5	0.298
Baseline hear rate, beats/min	76.5 ± 10.0	79.9 ± 8.2	0.127
25(OH)D level, nmol/L	37.58 ± 11.45	65.90 ± 15.90	<0.001
SDNN, ms	150.04 ± 37.05	154.00 ±35.18	0.644
pNN50, %	20.28 ± 8.18	20.56 ± 10.07	0.893
rMSDD, ms	45.56 ± 16.87	61.90 ± 20.38	<0.001
SDANN, ms	138.91 ± 28.25	139.83 ± 34.98	0.900

*BMI, body mass index; BP, blood pressure; HRV, heart rate variability; rMSSD, Square root of the mean of sum of the square of differences between adjacent normal to normal interval; SDNN, standard deviation of normal to normal; SDANN, standard deviation of the average normal to normal intervals; pNN50, percentage of differences between adjacent RR intervals that are >50 ms*.

**Figure 1:**
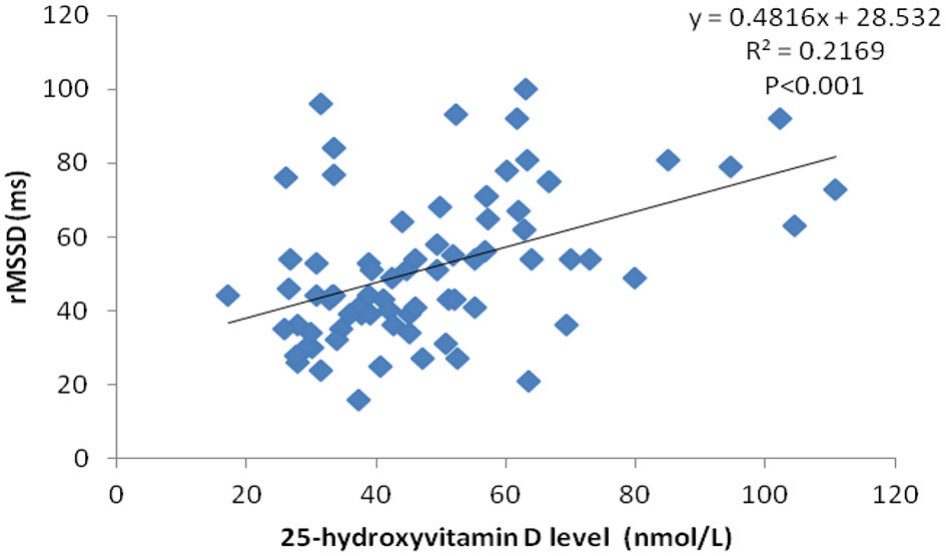
Correlation scatter plot between serum 25-hydroxyvitamin D level and rMSSD in VVS patients.

Serum 25-hydroxyvitamin D levels had a positive correlation with rMSDD values (*r* = 0.466, *p* < 0.05) using Pearson correlation analysis (Figure 1).

## Discussion

Our results suggest that hypovitaminosis D was common (at a rate of 60%) in children and adolescents with VVS at a rate of 60%. This group exhibited a significantly reduced rMSSD value, and the 25(OH)D levels in serum were positively correlated with rMSSD value. rMSSD reflects the parasympathetic activity of the heart, indicating a correlation between hypovitaminosis and the disturbance of cardiac autonomic nervous function, which may participate in VSS occurrence and progression among child and adolescent subjects. We speculate that vitamin D deficiency results in a decreased cardiac vagal tone which further leads to the augmentation in basal sympathetic activity. Increased baseline sympathetic activity, combined with modulation of the cardiovascular autonomic nervous system when assuming an upright position, makes the patients more predisposed to triggering the Bezold-Jarisch reflex and leading to a syncopal attack. However, we did not find that orther HRV indexes of sympathetic activity such as SDANN increased in vitamin D deficient children with VVS. Further studies with larger size are needed to confirm these findings.

Vitamin D deficiency is becoming a widespread nutritional disorder of epidemic proportions primarily caused by insufficient sunlight exposure, a high prevalence of obesity and poor eating habits (17). Previous reports showed that low levels of serum vitamin D were related to an increased susceptibility to many cardiovascular diseases (CVDs), such as orthostatic hypotension, hypertension, heart failure, and coronary artery disease (8, 9, 11, 12). Many studies found that vitamin D supplementation could also modulate the sympathetic nervous system of the heart in vitamin D deficient, but otherwise healthy young adults (10, 18).

The autonomic nervous dysfunction has been shown to participate in the pathogenesis of VVS in children and adolescents. HRV is measured by the exact fluctuations in the beat-to-beat interval and used for qualitative and quantitative evaluation of cardiac autonomic function. At present, HRV is a commonly recognized approach for the evaluation of cardiac autonomic function. Akçaboy et al. (19) studied 24-h HRV in children with VVS and reported that they exhibited a significantly increased SDNN compared to healthy control subjects. Zygmunt and Stanczyk (20) found that rMSSD and pNN50 (the proportion of the difference between adjacent normal to normal heart rate intervals >50 ms) values in syncopal children were lower than in healthy children. In addition, the children with syncope exhibited decreased high-frequency (HF) whereas increased low-frequency (LF) using the frequency-domain analysis. These results indicate a decreased vagal and increased sympathetic modulation in VVS patients.

The exact pathogenesis of VVS, however, remains unknown. VVS, postural tachycardia syndrome and orthostatic hypotension have similar etiologies. Studies in adults showed that hypovitaminosis D was independently correlated with orthostatic hypotension (12). Antiel et al. (13) found that the incidence of hypovitaminosis D in adolescent patients with POTS was higher than the normal adolescent population (30 vs. 14%, respectively) (21). Our study reported a possible association between hypovitaminosis D and VVS in children and adolescents. We found that hypovitaminosis D was more prevalent in VVS patients than in the healthy control group and may be involved in the pathological mechanism for VVS by affecting cardiac autonomic function. These findings are significant to the understanding of the VVS pathogenesis. Tønnesen et al. (18) found that young people (18–25 years old) with low serum vitamin D improved their autonomic dysfunction following 180-day vitamin D supplementation. Thus, supplementation of vitamin D is suggested to benefit the recovery from VVS in children and adolescents with hypovitaminosis D.

Our study has several limitations. We did not compare HRV of VVS patients with the normal controls. As mentioned above, there had been several studies including Akçaboy et al. (19) and Zygmunt and Stanczyk (20) which showed that HRV in children with VVS exhibited significant abnormalities compared with normal healthy children. Further research should include multi-center-based studies to identify vitamin D deficiency involvement in the regulation of cardiac autonomic functions in a wider range of patients with VVS. Other methods of autonomic nervous function assessment including frequency-domain analysis, quantitative Valsalva maneuver and heart rate changes with deep breathing in VVS patients are worthy of further investigation. Moreover, we failed to consider that the vitamin D levels were affected by exposure to the sun in different seasons, which might affect the accuracy of the results.

## Conclusion

Our data indicate that vitamin D deficiency may be correlated with cardiac autonomic dysfunction of pediatric vasovagal syncope. Further prospective large-scale studies are necessary to confirm these findings and better understand the role of hypovitaminosis D in the pathogenesis of autonomic dysfunction in patients with VVS.

## Data Availability Statement

The raw data supporting the conclusions of this article will be made available by the authors, without undue reservation.

## Ethics Statement

The studies involving human participants were reviewed and approved by The Ethics Committee of Peking University First Hospital. Written informed consent to participate in this study was provided by the participants' legal guardian/next of kin.

## Author Contributions

QZ and JD designed the study, drafted the initial manuscript, and reviewed and revised the manuscript. YS collected data, carried out the initial analyses, and reviewed and revised the manuscript. CZ contributed to the manuscript design and data analysis. JQ reviewed the manuscript, contributed to the literature overview, and data analysis. All authors approved the final manuscript as submitted and agree to be accountable for all aspects of the work.

## Conflict of Interest

The authors declare that the research was conducted in the absence of any commercial or financial relationships that could be construed as a potential conflict of interest.

## Abbreviations

VVS: vasovagal syncope
25(OH)D: 25-hydroxyvitamin D
HRV: heart rate variability
BMI: body mass index
BP: blood pressure
rMSSD: square root of the mean of sum of the square of differences between adjacent normal to normal interval
SDNN: standard deviation of normal to normal
SDANN: standard deviation of the average normal to normal intervals
ECG: electrocardiogram
SD: standard deviations.
